# Supplementary material for: Global hypo-methylation in a proportion of glioblastoma enriched for an astrocytic signature is associated with increased invasion and altered immune landscape
Source: eLife. 2022 Nov 22;11:e77335. doi: 10.7554/eLife.77335 (PMC9681209; doi:10.7554/eLife.77335)
Supplement: Figure 2—source data 1. [file elife-77335-fig2-data1.zip › Figure_2_source_data_1/Figure_2B/homerResults/motif12.similar.html]

motif12

## Information for motif12

A
G
T
C
C
G
T
A
A
C
T
G
C
G
T
A
A
C
T
G
C
G
T
A
A
C
T
G
A
C
T
G
A
G
T
C
A
G
T
C
  
Reverse Opposite:  

A
C
T
G
A
C
T
G
A
G
T
C
A
G
T
C
A
C
G
T
A
G
T
C
A
C
G
T
A
G
T
C
A
C
G
T
A
C
T
G
  

|  |  |
| --- | --- |
| p-value: | 1e-67 |
| log p-value: | -1.544e+02 |
| Information Content per bp: | 1.530 |
| Number of Target Sequences with motif | 89.0 |
| Percentage of Target Sequences with motif | 3.85% |
| Number of Background Sequences with motif | 1.9 |
| Percentage of Background Sequences with motif | 0.54% |
| Average Position of motif in Targets | 114.5 +/- 50.5bp |
| Average Position of motif in Background | 129.5 +/- 45.9bp |
| Strand Bias (log2 ratio + to - strand density) | 0.2 |
| Multiplicity (# of sites on avg that occur together) | 1.01 |
| Motif File: | file (matrix) reverse opposite |

### Similar de novo motifs found

|  |  |  |  |  |  |  |  |
| --- | --- | --- | --- | --- | --- | --- | --- |
| Rank | Match Score | Redundant Motif | P-value | log P-value | % of Targets | % of Background | Motif file |
| 1 | 0.779 | G T A C G T A C G C T A A C T G A T C G A T G C G A T C G A C T A T G C G A C T A T G C G A C T | 1e-39 | -90.179573 | 4.41% | 1.01% | motif file (matrix) |
